# Psychiatric emergencies of minors with and without migration background

**DOI:** 10.1007/s40211-016-0213-y

**Published:** 2016-12-13

**Authors:** Türkan Akkaya-Kalayci, Christian Popow, Thomas Waldhör, Dietmar Winkler, Zeliha Özlü-Erkilic

**Affiliations:** 1 Outpatient Clinic of Transcultural Psychiatry and Migration Induced Disorders in Childhood and Adolescence, Department of Child and Adolescent Psychiatry, Medical University of Vienna, Währinger Gürtel 18–20, 1090 Vienna, Austria; 2 Department of Child and Adolescent Psychiatry, Medical University of Vienna, Währinger Gürtel 18–20, 1090 Vienna, Austria; 3 Department of Epidemiology, Medical University of Vienna, Kinderspitalgasse 15/I, 1090 Vienna, Austria; 4 Department of Psychiatry and Psychotherapy, Medical University of Vienna, Währinger Gürtel 18-20, 1090 Vienna, Austria

**Keywords:** Psychiatric emergency, Children and adolescents, Migration background, Turkish children and adolescents, Serbian/Croatian/Bosnian children and adolescents, Psychiatrischer Notfall, Kinder und Jugendliche, Migrationshintergrund, Türkischsprachige Kinder und Jugendliche, Bosnisch/Kroatisch/Serbische Kinder und Jugendliche

## Abstract

**Background:**

The conditions of children and adolescents with migration background receiving emergency psychiatric care in Europe are not well known. Migrants usually attend regular psychiatric care less frequently than the autochthonous population. We therefore speculated that, being undertreated, they would be overrepresented among psychiatric emergency care patients.

**Methods:**

We retrospectively analyzed the records of 1093 minors aged 4‑18 years treated during a period of three years at the psychiatric emergency outpatient clinic of the Department of Child and Adolescent Psychiatry at the Medical University of Vienna.

**Results:**

More minors with migration background than natives consulted our emergency clinic. Most frequent reasons for referral were suicide attempts by Turkish patients, acute stress disorder in Serbian/Croatian/Bosnian and in Austrian patients. Psychiatric diagnoses like eating and personality disorders were mostly diagnosed in natives. We found gender specific differences between the groups.

**Conclusions:**

The reasons for these differences possibly relate to deficits of adequate mental health-care in Austria, to intercultural and intrafamiliar conflicts related to acculturation distress in the migrant population. Prospective longitudinal studies focusing on the utilization of mental health care by the migrant children and the impact of the migration background on their mental health are needed for improving adequate culture-sensitive mental-health care for this population.

## Zusammenfassung

**Grundlagen:**

In Europa ist die Situation von Kindern und Jugendlichen mit Migrationshintergrund, die an der psychiatrischen Akutversorgung behandelt werden, nicht ausreichend bekannt. Gewöhnlich besuchen Migranten reguläre psychiatrische Betreuung weniger häufig als die autochthone Bevölkerung. Daher spekulierten wir, dass sie wegen der Unterversorgung in der regulären psychiatrischen Betreuung in der psychiatrischen Akutversorgung überrepräsentiert sind.

**Methodik:**

Wir haben die Patientenakten von 1093 Minderjährigen zwischen 4–18 Jahren, die über drei Jahre in der Wiener Universitätsklinik für Kinder- und Jugendpsychiatrie an der psychiatrischen Akutambulanz behandelt wurden, retrospektiv analysiert.

**Ergebnisse:**

Mehr Minderjährige mit Migrationshintergrund als einheimische konsultierten unsere psychiatrische Akutambulanz. Die häufigsten Gründe für die Akutvorstellungen waren Suizidversuche bei Türkischsprachigen Patienten, akute Belastungsstörung bei den Bosnisch/Kroatisch/Serbischen und bei den österreichischen Patienten. Psychiatrische Diagnosen wie Essstörungen und Persönlichkeitsstörungen wurden überwiegend bei einheimischen Patienten diagnostiziert. Außerdem konnten wir Geschlechtsunterschiede zwischen den Gruppen erfassten.

**Schlussfolgerungen:**

Die Gründe für diese Unterschiede sind vermutlich durch Mangel an ausreichender psychiatrischer Gesundheitsversorgung der Migranten, durch Akkulturationsstress bedingte interkulturelle sowie intrafamiliäre Konflikte, verursacht. Daher sind prospektive Langzeitstudien, die sich auf den Einfluss des Migrationshintergrunds auf die psychische Gesundheit von Minderjährigen konzentrieren notwendig, um adäquate kultursensible Angebote für junge Migranten zu entwickeln.

## Background

Mental health disorders are commonly observed in all societies and age groups. Almost half of the psychiatric disorders begin in childhood and adolescence. The prevalence of psychiatric disorders in youth has steadily increased in the last decades and will probably continue to rise [1]. Detecting underlying causes seems of utmost importance because mental health problems may cause severe disability.

Poverty and migration background increase the risk of developing psychosocial disorders in children and adolescents [2, 3]. Cultural, familial, and religious differences, migration and acculturation related distress, economic, health and education related disadvantages, limited acceptance by the host population, and limited participation in common cultural activities are among the most important causes for personal disadvantages of the migrant population, increasing the risk of mental health disorders [2, 3], especially affective, anxiety, and psychosomatic disorders [4–7]. Limited language proficiency, and female gender further increase the experienced distress and the risk of developing affective disorders [8]. Restricted access to mental health care services will interfere with adequate medical treatment, and the risk of developing chronic disorders, affective, anxiety, psychosomatic disorders, and of substance abuse [9].

The prevalence of mental health disorders may be influenced by ethnicity. Turkish-speaking adults had the highest prevalence of depressive and/or anxiety disorders followed by Moroccans in a Dutch study [10]. Turkish speaking adolescents report more internalizing problems compared to their indigenous peers [10]. Increased acculturation stress, language problems, an unfamiliar local and social environment, low socioeconomic status and cultural dissimilarities may explain some of the health related differences between the migrant and the autochthonous population [5, 7, 11–13].

Although the prevalence of psychiatric problems may be higher in the migrant population, children and adolescents with migration background are disadvantaged in accessing mental health care [14] because migrants label mental health problems as disgraceful and tend to ignore them. Being less articulate and facing low cultural competence of mental health professionals, they may avoid attending mental health care, medication and psychotherapy [4, 5, 15–19]. This may lead to increased utilization of emergency psychiatric care [14, 17, 20–22].

Although Austria is one of the wealthiest countries in the world, mental health care for children and adolescents is insufficient because of structural/organizational problems and insufficient human resources [23]. This leads to insufficient psychiatric care and an increased risk of disabilities [23], especially for the migrant population [24]. The ethnic diversity of the Austrian population has rapidly increased in the last four decades. In 2010, 1,543,300 people with migration background lived in Austria, representing 18.6% of the total population. Particularly children and adolescents comprise a considerable proportion of the total migrant population. Based on the census of 2010, 582,000 or 36.9% of the migrant population were younger than 29 years, and 257,400 or 16.3% of the migrant population were children and adolescents aged less than 15 years [25]. 351,907 people with migration background lived in Vienna, representing 20.7% of the population in 2010. Two of the biggest migrant groups living in Vienna originate from Ex-Yugoslavia (6.8%, now Serbia/Croatia/Bosnia) followed by the Turkish speaking population (2.5%). Serbian/Croatian/Bosnian speaking people from Ex-Yugoslavia are now mostly in their second and third generation and probably better adapted to the Austrian cultural background, except for Bosnian refugees of the Balkan War in the early 90s when 100,000 people were killed or missing, and an equal amount of people, partly Muslims, fled from the country*.*

Due to the lack of data available on psychiatric health-care utilization, especially of the migrant population in Austria, we compared native Austrian children and adolescents with their peers originating from Turkey, and from former Yugoslavia and their reasons for consulting our emergency outpatient clinic. The objective of our study was to analyze differences concerning clinical reasons for referral and their background in the three study groups. We hypothesized from data in international literature that migrant children and adolescents would be overrepresented in psychiatric emergency care compared to indigenous peers, and that we would find differences in the reasons of referral, psychiatric diagnoses, in gender, and the referring persons [8, 26]. To our knowledge, this is the first study conducted in Europe explicitly comparing reasons for psychiatric emergency referrals of children and adolescents with and without migration background.

## Methods

We retrospectively analyzed data extracted from the medical paper files of all patients, aged between 4–18 years, treated at the emergency outpatients’ clinic of the Department of Child and Adolescent Psychiatry at the Medical University of Vienna between January 2008 and December 2010.

From the 1821 children and adolescents admitted to our emergency outpatient clinic during the study period, we only considered the data of 1718 patients aged between 4–18 years, and analyzed the files of 1093 patients, 800 native Austrian patients and 293 patients originating from Turkey (*n* = 130) and Ex-Yugoslavia (*n* = 163), excluding 625 patients with a different migration background. According to Statistic Austria, migration background is defined by at least one parent or grandparent not being born in Austria [25].

For patients having more than one reason for referral, we chose the most important reason. For patients who had more than one emergency consultation, only the first consultation was considered.

The Department of Child and Adolescent Psychiatry in Vienna is involved in the medical care of more than half of the Viennese children and adolescents with mental health problems; our study sample is therefore representative for the Viennese population.

We designed a specific data sheet for our retrospective data analysis. It summarizes patient history, socio-demographic data, like ethnicity, age, sex, reasons for referral, diagnoses, first attendance at our clinic, and referring person for the present contact. The data sheet contains 95 variables as we collected quite detailed information of the patients.

We used SPSS 20 software [27] for analyzing our data including descriptive methods, unpaired t‑tests for analyzing differences between means, and chi-square statistics for analyzing differences of categorical variables. A *p* value ≤ 0.05 was considered to indicate statistical significance.

The Ethics Committee of the Medical University of Vienna approved the study protocol.

## Results

### Study sample

More than half (53.4%, *N* = 918) of the patients consulting our emergency clinic had a migration background, 46.6% (*N* = 800) were native Austrians, 9.5% originated from former Yugoslavia, 7.6% from Turkey, and 36.4% from other countries like India, Pakistan, Iran, Iraq, Afghanistan, Egypt etc.

Significantly more female patients (59.2%) were treated at the emergency outpatients’ clinic: 56.4% females among the Austrian, 71.5% among Turkish, and 63.2% among Serbian/Croatian/Bosnian patients (χ^2^ = 11.9; *p* = 0.003).

The mean age of the patients was 14.6 (SD = 3.0, range 4–18) years with the majority being between 12–18 years old (83.6%). Mean ages of Austrian (14.6 years), Turkish (15.1 years), and Serbian/Croatian/Bosnian children (14.1 years) were similar. Female patients were significantly older than males (14.97 ± 2.9 (SD) vs. 14.0 ± 3.2 (SD) years, t = 123.6, *p* < 0.001).

The majority of the patients (60.3%) had no previous psychiatric treatment, 57.2% of the Austrian, 70.8% of the Turkish and 66.9% of the Serbian/Croatian/Bosnian patients attended the psychiatric emergency clinic for the first time (χ^2^ = 12.0; *p* = 0.002). The remaining patients had already consulted our emergency clinic more than once because of acute problems.

### Reasons for referral

#### Reasons for referral and gender

The most frequently observed reasons for acute consultation (Table 1) were acute stress disorder (20.3%), behavioral problems (13.9%) and attempted suicide (13.4%). There were significant gender differences: female patients were most frequently seen with acute stress disorder (22.9%), suicide attempts (17.2%), or suicidality (10.0%), males for behavioral problems (24.9%), acute stress disorder (16.4%), or anxiety disorders (11.0%, χ^2^ = 125.4, *p* < 0.001).

**Table 1:** Reason for referrals by Native Austrians, Serbian/Croatian/Bosnian and Turkish speaking migrants

	Native Austrians^a^ (*N* = 800)				Serbian/Croatian/Bosnian speaking^b^ (*N* = 163)				Turkish speaking^c^ (*N* = 130)			
Reasons for referral	Female		Male		Female		Male		Female		Male	
	*N*	%	*N*	%	*N*	%	*N*	%	*N*	%	*N*	%
Psychotic disorders	2	0.4	7	2.0	1	1.0	2	3.3	0	–	2	5.4
Alcohol intoxication	5	1.1	7	2.0	0	–	0	–	0	–	0	–
Drug intoxication	12	2.7	16	4.6	5	4.9	3	5.0	0	–	0	–
Suicide attempt	66	14.6	23	6.6	23	22.3	4	6.7	22	23.7	8	21.6
Suicidal ideation	53	11.8	42	12.0	5	4.9	2	3.3	7	7.5	0	–
Self-harming behaviour	29	6.4	6	1.7	4	3.9	3	5.0	5	5.4	1	2.7
Acute stress disorder	110	24.4	57	16.3	20	19.4	11	18.3	18	19.4	6	16.2
Depressive episode	33	7.3	22	6.3	2	1.9	3	5.0	9	9.7	7	18.9
Anxiety/panic disorders	41	9.1	37	10.6	10	9.7	6	10.0	11	11.8	6	16.2
Child abuse	0	–	2	0.6	1	1.0	1	1.7	1	1.1	0	–
Eating disorders	37	8.2	7	2.0	5	4.9	3	5.0	2	2.2	0	–
Pain	21	4.7	16	4.6	6	5.8	7	11.7	10	10.8	3	8.1
Behavioural problems	25	5.5	93	26.6	10	9.7	14	23.3	6	6.5	4	10.8
School refusal	7	1.6	10	2.9	7	6.8	1	1.7	0	–	0	–
Sexual abuse	10	2.2	4	1.1	4	3.9	0	–	2	2.2	0	–
Total	451	100	349	100	103	100	60	100	93	100	37	100

^a^(X^2^ = 114.343; df = 14; *p* < 0.001)

^b^(X^2^ = 19.180; df = 13; *p* = 0.118)

^c^(X^2^ = 13.465; df = 11; *p* = 0.264)

The above-mentioned significances refer to all reasons for acute referrals

#### Reasons for referral by three study groups

Austrian children and adolescents were predominantly referred for acute stress disorder (20.9%), behavioral problems (14.8%), or suicidal ideation (11.9%), Turkish patients for attempted suicide (23.1%), acute stress disorder (18.5%), or anxiety/panic disorders (13.1%), and Serbian/Croatian/Bosnian children for acute stress disorder (19.0%), suicide attempts (16.6%), and behavioral problems (14.7%). The differences between the three study groups concerning their major reason for an acute referral were highly significant (χ^2^ = 72.4; *p* < 0.001).

We found significant gender differences (χ^2^ = 114.3, *p* < 0.001) in the Austrian group but not in the groups with migration background concerning the primary reason of referral (Table 1).

### Psychiatric diagnoses

In 68.6% of the patients a psychiatric diagnosis was assigned (Table 2). In the remaining patients the presented problems did not justify a psychiatric diagnosis or the attending physician did not assign a specific diagnosis.

**Table 2:** ICD 10 diagnosis by Native Austrians, Serbian/Croatian/Bosnian and Turkish speaking migrants

ICD 10 Diagnosis	Native Austrians^a^ (*N* = 573)				Serbian/Croatian/Bosnian speaking^b^ (*N* = 97)				Turkish speaking^c^ (*N* = 80)			
	Female		Male		Female		Male		Female		Male	
	*N*	%	*N*	%	*N*	%	*N*	%	*N*	%	*N*	%
F 1	10	3.1	5	2.0	2	3.3	1	2.7	1	1.7	2	10.0
F 2	11	3.4	15	6.0	2	3.3	6	16.2	1	1.7	1	5.0
F 3	85	26.4	42	16.7	14	23.3	5	13.5	15	25.%	3	15.0
F 4	118	36.6	82	32.7	28	46.7	7	18.9	31	51.7	6	30.0
F 5	31	9.6	1	0.4	4	6.7	3	8.1	3	5.0	0	–
F 6	29	9.0	3	1.2	1	1.7	1	2.7	1	1.7	0	–
F 7	1	0.3	3	1.2	1	1.7	3	8.1	0	–	1	5.0
F 8	5	1.6	15	6.0	1	1.7	1	2.7	1	1.7	1	5.0
F 9	32	9.9	85	33.9	7	11.7	10	27.0	7	11.7	6	30.0
Total	322	100	251	100	60	100	37	100	60	100	20	100

*F 1* Mental and behavioral disorders due to psychoactive substance use, *F 2* Schizophrenia, schizotypal and delusional disorders, *F 3* Mood (affective) disorders, *F 4* Neurotic, stress-related and somatoform disorders, *F 5* Behavioral syndromes associated with physiological disturbances and physical factors, *F 6* Disorders of adult personality and behavior, *F 7* Mental retardation, *F 8* Disorders of psychological development, *F 9* Behavioral and emotional disorders with onset usually occurring in childhood and adolescence

^a^(X^2^ = 95.244; df = 8; *p* < 0.001)

^b^(X^2^ = 16.333; df = 8; *p* = 0.038)

^c^(X^2^ = 13.736; df = 8; *p* = 0.089)

The above-mentioned significances refer to all diagnoses according ICD-10

The most frequently assigned diagnoses were F 4 – neurotic, stress-related and somatoform disorders (24.9%) followed by F 3 – affective disorders (15.0%) and F 9 – behavioral and emotional disorders (13.4%).

#### Psychiatric diagnoses and gender

We observed gender differences for ICD-10 defined diagnostic groups (χ2 = 108.0, *p* < 0.001): the most frequently observed diagnostic groups were F 4 (neurotic, stress-related and somatoform disorders, 36.6%), F 3 (affective disorders, 26.4%), and F 9 (behavioral and emotional disorders, 9.9%) in female patients; and F 9 (behavioral and emotional disorders, 33.9%), F 4 (neurotic, stress-related, and somatoform disorders, 32.7%), and F 3 (affective disorders, 16.7%) in males.

#### Psychiatric diagnosis, migration background and gender

We observed gender differences for ICD-10 diagnoses in the Austrian (X^2^ = 95.244; df = 8; *p* < 0.001) and Serbian/Croatian/Bosnian population (X^2^ = 16.333; df = 8; *p* = 0.038) but not in the Turkish (X^2^ = 13.736; df = 8; *p* = 0.089) population: In Serbian/Croatian/Bosnian and Austrian males the diagnosis, F 9 (behavioral and emotional disorders 27.0% vs. 33.9%), and in Serbian/Croatian/Bosnian and Austrian girls, the diagnosis, F 4 (neurotic, stress-related and somatoform disorders, 46.7% vs. 36.6%), were most frequently assigned. In Turkish males and females, the diagnosis, F 4 (neurotic, stress-related and somatoform disorders, 30.0 vs. 51.6%) was the predominantly assigned diagnosis (Table 2).

Contrary to the Austrian (5.6%) and Serbian/Croatian/Bosnian patients (7.2%), the diagnosis of eating disorders (F 50) was only rarely assigned to the Turkish population (3.8%). Personality disorders (F 60) were more frequently diagnosed in Austrian (5.6%) than in Turkish (1.3%) and Serbian/Croatian/Bosnian patients (4.1%).

### Referring persons

Patients were most frequently referred to our emergency clinic by their social network (45.5%), 15.6% of the patients were referred by physicians, 12.4% by various institutions, and 9.1% of the patients came on their own, and in 17.6%, the referring person was not known.

There were differences among the three groups concerning the referring persons: Austrian and Serbian/Croatian/Bosnian patients were mostly referred by their social network (48.0% vs. 50.9%), followed by physicians (15.0% vs. 20.2%). In the majority of the Turkish patients, the referring person was unknown (50.8%), followed by persons from the social network (23.1%; χ^2^ = 122.5; *p* < 0.001).

## Discussion

One of the main findings of our study was that children and adolescents with migration background were largely overrepresented at our psychiatric emergency outpatients’ clinic. This result holds, even considering that the number of children in the migrant, and especially in the Turkish population is larger than in native Austrians (about the double, 1.3 vs. 2.5). Similar to previous studies, the majority of the patients consulted emergency psychiatric care for the first time [28]. In our study, more children and adolescents with migration background (about 2/3) compared to native Austrian children (50%) presented at the psychiatric emergency clinic for the first time.

There are possibly multiple causes responsible for these results: although there is a comprehensive insurance system for all people living in Austria, the lack of adequate, cultural and language competent diversity-care leads to diminished utilization of regular mental health care services by children and adolescents with psychic problems and migration background. In addition, culture and shame-related fears of psychiatric disorders might reduce the readiness to consult regular psychiatric services. Consequently the patients will consult emergency mental health care when the problems become overwhelming [14, 24].

The significant gender differences – primarily females – for consulting emergency psychiatric care might be explained by the fact that especially adolescent girls with migration background were more affected by problems related to intrafamiliar conflicts and acculturation stress [21, 23]. Adolescent girls are more strictly controlled by their families and were reported to get less psychological care compared to their male peers [20, 21, 26]. In addition, male youths frequently present externalizing disorders leading to problems earlier in life, psychiatric referrals and treatment whereas internalizing disorders, more predominant in (adolescent) girls, are more easily neglected.

Another major finding of our study was that Serbian/Croatian/Bosnian and Turkish speaking children and adolescents were referred more frequently than natives for suicide ideation and attempts. Comparing similar studies in youth [29], suicidality was the most frequently reported reason for referral to emergency clinics in other countries. In our sample this was only observed in Turkish children who probably suffer more from acculturation stress [2, 21] because of the larger cultural and religious differences between the culture of origin and the dominant host culture [30], and probably because the Serbian/Croatian wave of immigration started about 10–15 years earlier than the Turkish wave in Austria, enabling nearly a whole generation to adapt to the (probably less different) host culture. In addition, German language problems which are more pronounced in the Turkish compared to the former Yugoslavian population [24], and the reduced availability of qualified Turkish speaking interpreters (often the siblings) may be held responsible for more data lacking data in the Turkish population.

We also found differences concerning the reasons for referral: Austrian children consulted the emergency psychiatric outpatient clinic most frequently for acute stress and behavioral disorders. Turkish children and adolescents were most frequently referred for suicidal attempts and acute stress disorder, and Serbian/Croatian/Bosnian youth were most frequently referred for acute stress disorder followed by suicidal attempts. There were significant differences in the reasons of referral not only between native and migrant patients and also between the two migrant groups. In agreement with our results, other studies reported that for minors with migration background the usual source of referral to the mental health services was medical experts, teachers or social workers [31].

The distribution of personality disorders was different in the three populations. This may be attributed to the fact that (Austrian) patients with this disorder usually are well-known recurrent clients at the emergency clinic, and when they get into suicidal crises, they will be attributed the primary diagnosis whereas in the migrant population who mostly appear for the first time at the emergency service, this diagnosis is not attributed with lightly, especially in younger adolescents. In addition, the fact that many migrant patients consulted the emergency clinic because of suicidal ideation or attempts may possibly be explained by the larger differences in values between the culture of origin and the host culture [2, 31], leading to emotional distress, and especially Turkish patients do not possess other exit strategies than a suicide attempt to escape from intrafamiliar, intra- and interpersonal conflicts [2, 5, 21, 32].

Our observation of no significant gender differences in psychiatric diagnoses in Turkish compared to a female overrepresentation in Austrian patients is confirmed by previous studies e. g. [33].

## Conclusion

Migrants can be considered a vulnerable group in the health-care systems [14, 34]. Numerous structural, political, socio-cultural as well as migration-specific factors lead to reduced utilization of health-care resources [11, 14] in Austria and in other European countries [35]. In addition, the health-care needs of children and adolescents with migration background are different compared to their autochthonous peers [34, 36]. Offering adequate health-care services to young people with migration background is difficult because specifically trained, language-proficient medical staff are not ubiquitously available.

Although the number of immigrant children and adolescents living in European countries is high, studies analyzing mental health problems of migrant children and adolescents are rare [7, 14]. The majority of the available studies focus on physical illnesses like infectious diseases or dental health problems [34]. As a consequence of these deficits, culture-specific symptoms of patients with migration background may be misinterpreted and may lead to inadequate diagnoses and treatment [14, 37]. This is especially true for psychiatric disorders [37]. Therefore, growing awareness of the different cultural background and values, on the diagnosis and treatment of migrant mental health disorders, and especially longitudinal studies are needed in order to provide adequate, appropriate, and preventive patient-centered care to this population and to decrease emergency care consultations [7, 13, 14, 33, 36, 38]. In addition, preventive measures should be established particularly in the natural settings of young people, for example in schools [39].

## Limitations

The present study is based on a retrospective analysis; therefore missing data could not be replenished. During an acute crisis, the patient history may not be ascertained in detail, therefore data about socio-demographic background were missing in more than half of the patients. More data were missing in patients with migration background probably because of insufficient language skills of the patients, their parents, and the caring physicians. Missing data are a relevant problem because of a possible selection bias. We analyzed psychiatric emergencies of children and adolescents originating from Turkey and former Yugoslavia living in Vienna. Therefore, the results cannot be generalized to other groups of migrants living in Austria.
